# Using arthroscopy combined with fluoroscopic technique for accurate location of the bone tunnel entrance in chronic ankle instability treatment

**DOI:** 10.1186/s12891-021-04165-0

**Published:** 2021-03-18

**Authors:** Shijun Wei, Dongsheng Fan, Fang Han, Ming Tang, Changwang Kong, Feng Xu, Xianhua Cai

**Affiliations:** 1 Department of Orthopaedics, General Hospital of Central Theater Command (Wuhan General Hospital of Guangzhou Command, previously), NO. 627, Wuluo Road, Wuhan, 430030 Hubei Province People’s Republic of China; 2 The First Clinical Medical School of Southern Medical University, Guangzhou, Guangdong Province People’s Republic of China; 3 Department of Orthopaedics, The Second Affiliated Hospital of Inner Mongolia Medical University, Hohhot, Inner Mongolia Province People’s Republic of China; 4 Department of Nutrition, General Hospital of Central Theater Command (Wuhan General Hospital of Guangzhou Command, previously), Wuhan, Hubei Province People’s Republic of China

**Keywords:** Chronic ankle instability, Ankle lateral ligament reconstruction, Semitendinosus autograft, Arthroscopy

## Abstract

**Background:**

Minimally invasive reconstruction techniques are used for anatomical ligament construction of the lateral collateral ligament complex of the ankle, but the two key elements, the bone tunnel and the appropriate graft tension, for the identification of the anatomic location during the surgery are not clearly stated.

**Methods:**

The patients with chronic ankle instability who received arthroscopic anatomic lateral ligament complex reconstruction were retrospectively analyzed. The anatomical location of the bone tunnel was performed under arthroscopy combined with fluoroscopy for accurate location of the bone tunnel entrance. The graft tension and routing were controlled under arthroscopic visualization. The clinical outcomes were assessed using the Karlsson-Peterson score, Sefton articular stability scale, and Visual Analogue Scale (VAS). The complications were recorded during the follow-up.

**Results:**

**A total of 18 patients were enrolled in this study.** The mean follow-up was 33.33 ± 3.69 (range from 24 to 36) months. No patient had recurrence of ankle instability after the operation. According to the Sefton articular stability scale, 94.5% of the patients had excellent/good function. The mean value of the anterior drawer tests and the talar tilt angle examination were decreased. The mean of the Karlsson-Peterson score and the Visual Analogue Scale(VAS) score were both improved significantly.

**Conclusions:**

The anatomic reconstruction of the ankle lateral ligament complex to treat chronic ankle instability using the arthroscopy combined with the fluoroscopic technique could improve the clinical functions, satisfaction, and reduced pain of patients.

## Background

Lateral ankle sprains are the most common ankle sports injury, and approximately 20–30% of patients may develop chronic ankle instability [1]. Chronic ankle instability resulting in reduced level of physical function or decreased quality of life is a significant health problem for patients [2]^.^ Besides, it could cause a significantly increased risk of post-traumatic arthritis in the ankle joint [1, 3–5]. Standard operative procedures for chronically unstable ankles included anatomical repair, non-anatomic, and anatomic reconstruction [6]. The non-anatomic lateral ankle ligament reconstruction procedure has the advantages of simplicity and effectiveness, but most patients after this procedure were reported to have a high incidence of joint stiffness and osteoarthritis [7, 8]. The Broström-Gould procedure, which is the injured ligament repaired by using local tissue, has been considered as the gold standard of operative treatment for chronic ankle instability [9, 10]. It is an effective anatomic repair procedure in which the damaged anterior talofibular ligament (ATFL) and calcaneofibular ligament (CFL) are repaired and strengthened by the inferior extensor retinaculum to provide an extra ankle stabilization [11]. The key to the success of this procedure depends on the quality of the ligament remnant and inferior extensor retinaculum [7, 12]. The anatomic reconstruction procedure is a more suitable approach for patients with poor quality of tissue and remnant, long-standing ankle instability, and severe local tissue scarring. Studies have demonstrated that anatomic reconstruction leads to better long-term effectiveness than non-anatomic reconstruction [1–3, 12].

In recent years, minimally invasive operative techniques, such as percutaneous or arthroscopic techniques, have been increasingly used in anatomic reconstruction [13]. The arthroscopic reconstruction techniques have gained popularity due to their ability in precise positioning which reduces surgical aggression and shortens the rehabilitation period [12, 14]. The two key elements in the arthroscopic anatomical reconstruction technique are the identification of the anatomic landmarks of the bone tunnel and the appropriate graft tension. There have been several arthroscopic reconstruction techniques used in the last decade with great variations in the construction of bone tunnels and grafts. However, most studies have not clarified anatomical ligaments reconstruction [14, 15]. The identification of anatomical landmarks and their precise distance from the ATFL and CFL footprints is essential to allow surgeons to accurately determine the anatomic location of these footprints and is very important in the repair or reconstructive surgery for CAI [14]. The fibular obscure tubercle (FOT) is located between the footprint center of the ATFL and CFL and is also located close to the intersection point of the ATFL and CFL [15]. Accordingly, these findings suggest that FOT can serve as a reliable and clinically relevant landmark to locate the origin of the ATFL and CFL for percutaneous minimally invasive procedures to treat CAI. However, it is difficult to accurately locate the calcaneal point of the CFL under the arthroscopy, so fluoroscopic positioning could be used in combination with the arthroscopic technique for accurate location of the CFL reconstructed calcaneal tunnel entrance. For autografts, fibula tendon, gracilis tendon, and semitendinous tendon are normally chosen for ligament reconstruction in CAI. The fibula tendon plays an important role in the stability of the ankle joint. Therefore, the fibular tendon should be avoided in the ligament reconstruction of CAI. The reconstruction of ATFL and CFL requires that the tendon has a sufficient diameter, so it is most ideal to choose the semitendinous tendon as a graft in this study.

This retrospective study evaluated the radiographic and clinical outcomes of anatomic reconstruction ankle lateral ligament complex using arthroscopy combined with the fluoroscopic technique for the treatment of chronic ankle instability.

## Methods

### Patients

A total of 18 patients with chronic ankle instability were treated in our hospital from December 2015 to February 2017 and retrospectively reviewed in this present study.

### Inclusion and exclusion criteria

The main criteria for the patients with chronic ankle instability included in this study were: (1) recurrent ankle sprains with ineffective conservative treatments included strength training of the fibularistertius muscle and proprioceptive training for 3–6 months; (2) a talar tilt angle of around 15° or more and positive anterior drawer tests on stress radiographs [16]; (3) the anatomical structure formed by ATFL and CFL on MRI was unclear; (4) severe ATFL remnant scarring, poor tissue elasticity, and inability to achieve successful suture repair on ankle joint arthroscopy; and (5) the willingness of younger patients (less than 50 years old) to recover their athletic ability. The main exclusion criteria were: (1) an inconsistent diagnosis with chronic ankle instability or inclusion standards; (2) the abnormal bone structure of the distal end of the fibula on X-ray and CT scans; (3) patients with low sports requirements.

Before surgery, standing weight-bearing lateral, ankle joint inversion stress anteroposterior/anterior drawer stress radiographs, 3D reconstruction CT scan, and MRI scan were performed. This study was approved by the Institutional Review Board (IRB) of the hospital. The consent form had been signed before the surgery.

### Operative methods

Since 2015, the anatomic reconstruction of the ankle lateral ligament complex has been performed using **arthroscopy combined with the fluoroscopic technique** for patients with chronic ankle instability.

#### Debridement and preparation of the bone tunnels

**Fig. 1:**
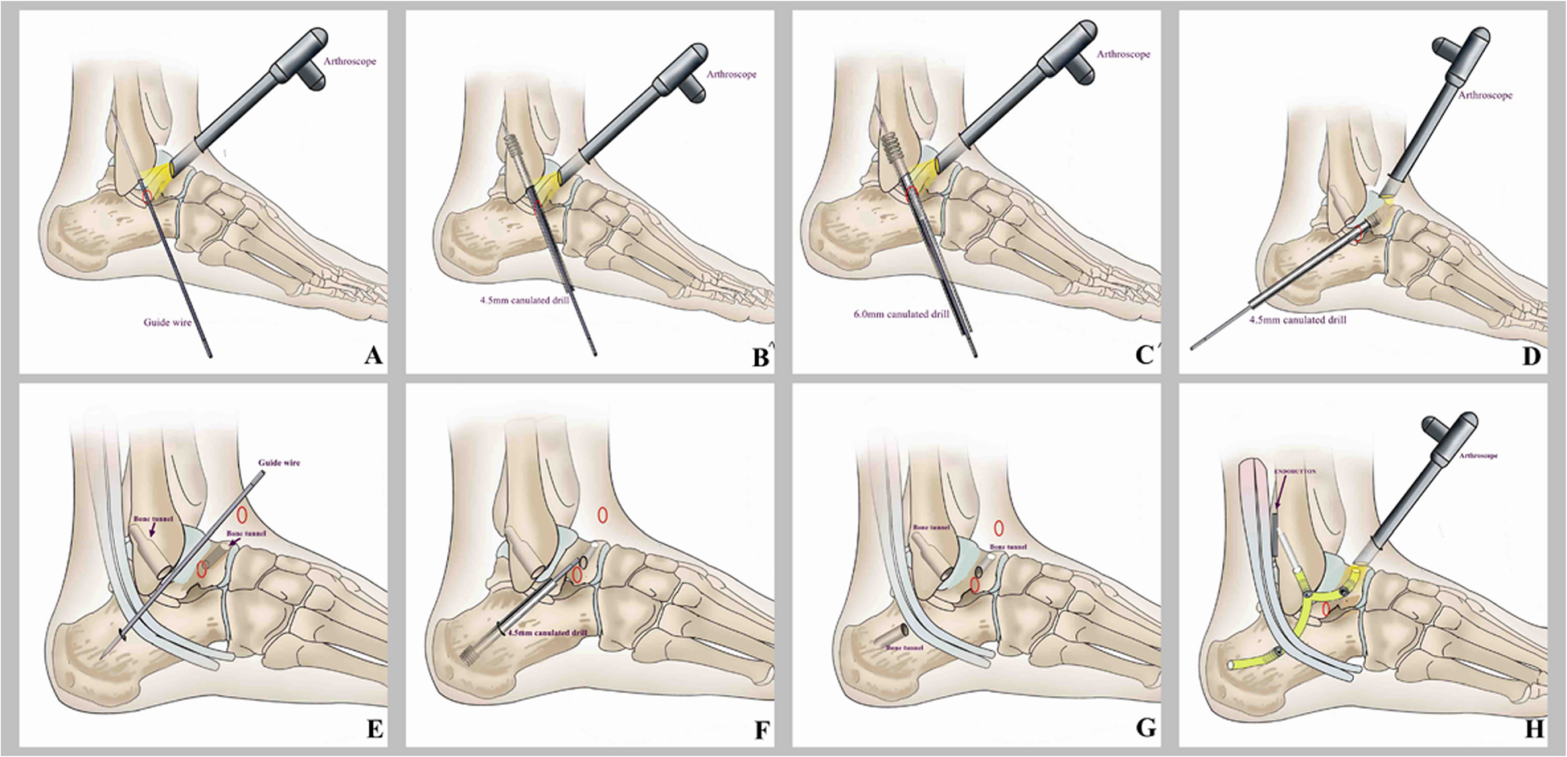
Schematic diagram of surgery. **a, b, c** The fibular bone tunnel preparing. **d** The talar bone tunnel preparing. **e** and **f**. The calcaneal bone tunnel preparing. **g** and **h** Tendon graft placement and fixation

**Fig. 2:**
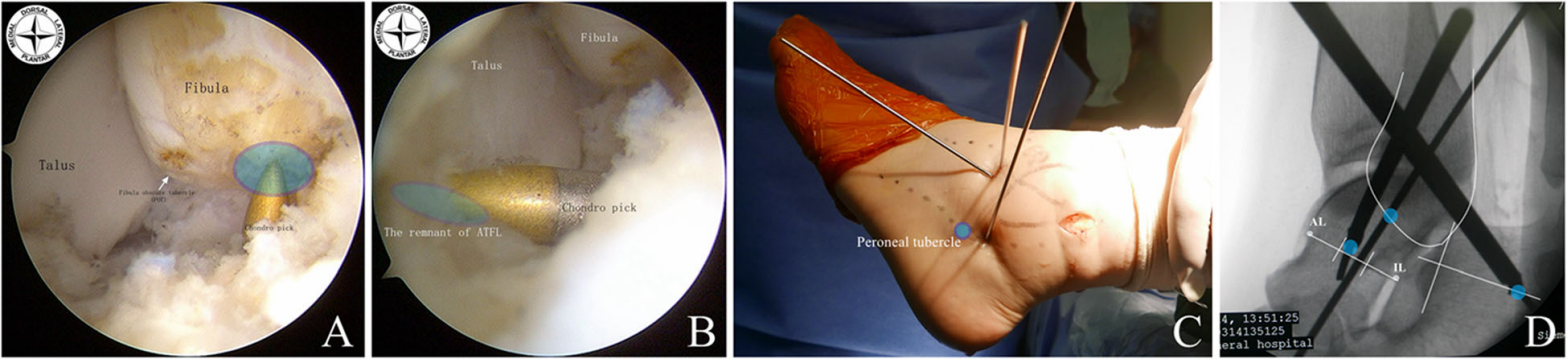
Locate the anatomic reconstruction point of bone tunnel. **a:** The anatomic reconstruction point of the fibular bone tunnel (5 mm lateral away from FOT) under arthroscopic visualization**; b:**The anatomic reconstruction point of the talar bone tunnel **(**the center of the ATFL talar footprint area) under arthroscopic visualization; **c**: The anatomic reconstruction point of the calcaneal bone tunnel; **d**: Fluoroscopic evaluation with lateral view of ankle before the tunnel drilling: the anatomic reconstruction point of the fibular bone tunnel locatedat 10 mm upper of the tip of distal fibula (blue point); Draw a line from the inferolateral (IL, white point) corner to the anterolateral (AL, white point) corner of the tall body, divide this line into three equal parts, and the entrance of the talar guide wire (blue point) should be located in the area above the middle part (corresponding to TOT); The entrance of the calcaneal guide wire should be located approximately 17 mm away from the vertical line at the midpoint of the posterior calcaneus articular surface

All patients undergoing this technique in our hospital had epidural anesthesia or spinal anesthesia. The patients were positioned supine with a pneumatic tourniquet on the root of the thigh. An ankle arthroscopic procedure was performed initially. The schematic diagram of the arthroscopic anatomical reconstruction of the lateral ankle ligament operative procedures is shown in Fig. 1. Anatomic landmarks are outlined on the skin, including medial and lateral malleoli, the anterior joint line, the tibialis anterior tendon (plantar flexion /dorsiflexion), peroneus tertius tendon (running along the superficial peroneal nerve), peroneal tendon (running along the sural nerve), ATFL and CFL ligaments are located. Skin preparation was performed in a standard approach. An Esmarch bandage was used to exsanguinate the leg, and the tourniquet is inflated. Firstly, a modified anteromedial (AM) portalis was established. The tibialis anterior tendon was pushed laterally when the anteromedial portal was made. So, it was more lateral than the classic locations to improve visualization of the lateral ankle gutter. The 30-degree 2.7 mm or 4.0mm arthroscopies were inserted to view the condition of the ATFL, an anterolateral portal (AL), and accessory anterolateral portal were established with the aid of the arthroscope. The medial and lateral gutter and anterior compartment were observed, and osteophytes and loose bodies are removed. The debridement of the osteochondral lesion was preferred. The quality of the ligament remnant was assessed with the probe. The lateral gutter was dissected carefully using a 2.9 mm end-cutting shaver. Visualization of the lateral gutter was significantly improved by full dorsiflexion of the ankle. The anterior-inferior tibiofibular ligament (AITFL) and the fibular footprint of the ATFL were identified. The Fibular obscure tubercle (FOT) is located adjacent to the footprint center of the ATFL and CFL. It is a key anatomic mark on the distal fibula. A guide wire was then introduced with a trajectory from anterior-inferior to posterior-superior at a 30° angle from the longitudinal axis of the fibula through the footprint center via the accessory anterolateral (acAL) portal. The lens of the arthroscopies were then rotated toward the lateral surface of the talus to confirm the footprint of the talarremnant of the ATFL which is just adjacent to the anterior edge of the lateral talar articular surface. We used a 45-degree microfracture awl to create a pilot hole in the center point of the footprint. Sometimes, the arthroscope could be introduced to the anterolateral portal to improve the viewing area on the talar footprint of the ATFL. Another guide wire was then introduced via the acAL portal, this guide wire was visualized entering into the center of the talar footprint of the ATFL and passing through the talus 1 cm from the anterior of the medial malleolus. Care was taken not to damage the medial joint surface of the talus or the talonavicular joint at the exit point of the talus. The third guide wire was introduced percutaneously above the tuberculum ligament calcaneofibular (TLC) in the direction ofthe posterior-inferior area of the calcaneal medial wall. This guide wire should avoid blood vessels, nerves, and tendons on the medial side of the calcaneus. At this stage, a 4 mm stab incision was established on the TLC of the lateral wall of the calcaneus. This key point was usually located approximately 5 mm posterior and proximal to the peroneal tubercle. Fluoroscopy is applied to confirm the locations of these guide wires. At the lateral view of fluoroscopy, the position of the entrance of the fibular guide wire was located at the corner of the articular surface of the distal fibula (corresponding to FOT). A line was drawn from the inferolateral (IL) corner to the anterolateral (AL) corner of the talar body, and this line was divided into three equal parts. The entrance of the talar guide wire should be located in the area above the middle part (corresponding to TOT). The entrance of the calcaneal guide wire was located approximately 17 mm away on the vertical line at the midpoint of the posterior calcaneal articular surfaceas shown in Fig. 2.

**Fig. 3:**
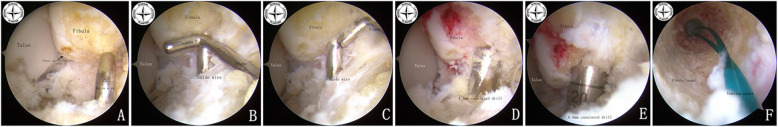
Drill the fibular bone tunnel under arthroscopic visualization. The guide wire was introduced at the midpoint of the ATFL fibular foot print area. The safe distance of the guide wire between the articular surface and the lateral wall was confirmed by the probe **a, b, c**. A 4.5 mm cannulated drill was introduced via the guide wire to create a full-thickness tunnel in the fibula while the length of the tunnel was measured **d**. A cannulated drill with a diameter of 6.0 mm was then introduced via the guide wire to deepen the tunnel to a depth of 20 mm **e**. Arthroscopic view of the fibular bone tunnel **f**

**Fig. 4:**
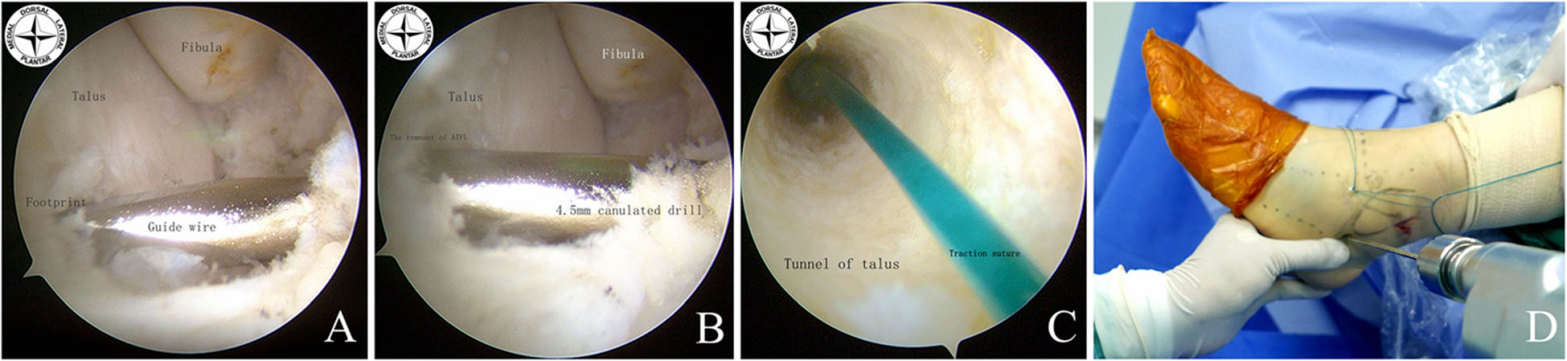
Drill the talar and calcaneal bone tunnel. The guide wire was introduced at the midpoint of the ATFL talar footprint area. There is a narrow bare area between the anterior edge of the lateral talar articular surface and the remnant of ATFL **a**. A 4.5 mm cannulated drill was introduced via the guide wire to create a full-thickness tunnel in the fibula while the length of the tunnel was measured **b**. Arthroscopic view of the talar bone tunnel **c**. A 4.5 mm cannulated drill was introduced via the guide wire to create a full-thickness tunnel in the calcaneus **d**

A 4.5 mm cannulated drill bit (Smith & Nephew Advanced Surgical Devices, Andover, MA) was introduced and prepared a 4.5 mm full-thickness fibular bone tunnel following the guide wire via the acAL portal. The length of the fibular bone tunnel could be estimated using the measurement scale on the drill bit at the time of posterior cortical breakthrough. After measuring the fibular bone tunnel length (approximately 35–40 mm), a 6.0 mm cannulated drill bit is then used to prepare the fibular bone socket to 2cm depth following the guide wire. In practice, before drilling the fibular bone tunnel, the safe distance between the guide wire and lateral cortical and medial articular surface of the distal fibula should be measured with a probe. The break of lateral cortical and medial articular surface of the distal fibula was avoided by these calculations. If the lateral wall of the fibular tunnel was damaged, an anchor near the entrance side can be used to fix the tendon graft (Fig. 3). The 4.5 mm cannulated drill bit was used to prepare a full-thickness talar bone tunnel following the guide wire via acAL portal. Similarly, the safe distance between the guide wire and lateral cortex of the talar neck were measured with the probe. The locations and bony walls of the two bone tunnels were confirmed by arthroscopy. The 4.5 mm cannulated drill bit was used to prepare percutaneously a full-thickness calcaneal bone tunnel following the guide wire. Three PDS sutures (#2 polydioxanone suture) which were used for tracing the tendon graft were passed through the bone tunnels using the Keith needle (Fig. 4)

.

#### Tendon graft preparation and fixation

**Fig. 5:**
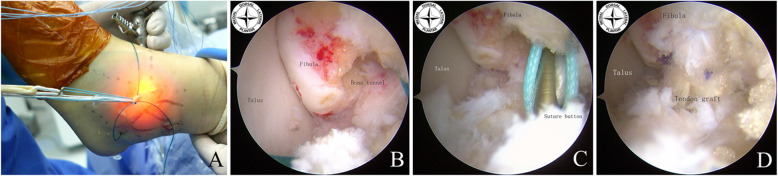
Implantation of the tendon graft and fixation of the fibular side. The Keith needle with the PDS suture (2 polydioxanone suture) was passed through the bone tunnel **a**, Under arthroscopic visualization, the ENDOBUTTON plate and the folded side of Y-shaped tendon graft were introduced into the bone tunnel using the PDS suture **b, c**, and then the end of the tendon graft was pulled back to make sure the ENDOBUTTON plate attached to the posterior cortex of the fibula **d**

**Fig. 6:**
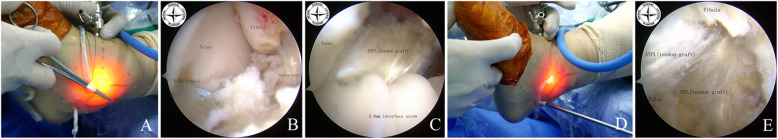
Implantation of the tendon graft and fixation of the talar and calcaneal side. The two strands of the tendon graft were introduced into the calcaneus and tibia bone tunnel, respectively **a, b**. To maintain the ankle joint in the neutral position and the proper tension at the end of the tendon graft, a 5 × 23 mm interference screw was placed on the calcaneus side, 5 × 13 mm interference screw was placed on the talar side for fixation **c, d**. The tension in the tendon graft was evaluated under arthroscopic visualization **e**

The semitendinosus tendon autograft was harvested from the ipsilateral knee joint and a Y-shaped tendon graft was prepared for anatomical reconstruction of lateral ankle ligament complex (ATFL and CFL). The free end of the tendon graft was tubularized with a locking whipstitch using a # 2 Fiber Wire suture. The tendon graft was passed through the polyester loop of the 15 mm ENDOBUTTON (Smith & Nephew, London, UK) and then folded to be a Y-shaped tendon graft. A # 2 absorbable suture was used to make a marked stitch at a 2 cm distance of the folded end of the graft. The PDS traction suture was used to pull the ENDOBUTTON and the Y-shaped tendon graft into the fibular bone tunnel until the ENDOBUTTON passed through the posterior fibular cortex, which was then flipped by counter traction on the end of the tendon graft so that it is seated directly on the posterior cortex of fibula. In practice, when the mark of the folded end of the tendon graft passes through the entrance of the fibular bone socket, this indicates that the button plate had passed through the posterior cortex and can be flipped. This step could be checked with the posterior small incision of the fibula (Fig. 5). When the ENDOBUTTON was completely seated on the fibular posterior cortex, the two strands of the tendon graft were shuttled out through the calcaneal and talar bone tunnel using the PDS traction sutures, respectively. In practice, the strand of the calcaneal side should be pulled into the bone tunnel firstly and ensured that it passes through under the peroneal tendon. The derotation of the strands could be achieved using a blunt trocar or forceps and maintaining the graft tension. The two strands of the tendon graft were conditioned by moving the ankle in flexion and extension 20 times under traction on the end of the tendon graft. Then the ankle was held in a neutral position with appropriate adjustment of the tension of the tendon graft. The talar fixation of the tendon graft was performed using a 5 × 13 mm interference screw (Milagro; DePuySynthes, Raynham, MA), while the calcaneal fixation of the tendon graft was performed using a 5 × 23 mm interference screw. Sometimes, if the reliability of fixation of the tendon graft in the talar side was doubted; thus, another one 5 × 13 mm interference screw at the exit of the talar bone tunnel is used. Finally, the free ends of the tendon graft were cut at the exit of the bone tunnels (Fig. 6). The portal incisions were closed using 3–0 Proleene sutures.

### Postoperative management

After the operation, the ankle was immobilized in a neutral position using a short leg brace. The affected limb was elevated, and cryotherapy was applied. After the pain subsided 2 days postoperative, the patients were encouraged to flex their toes actively. Rehabilitation exercises including non-weight bearing dorsiflexion and plantar flexion were started 1 week after the operation. Two weeks after the operation, weight- bearing ankle motion exercises and walking with the use of an ankle brace or boot were encouraged. The patients could gradually resume jogging and low-impact activities after 3 months with the protection of an ankle brace. However, competitive sports were prohibited for the first 6 months postoperative.

### Clinical evaluation

The patients had received follow-up examinations at 1, 2, 3, 6, 12, 18, and 24 months postoperative, and the state of incision healing and relevant complications were recorded during the postoperative follow-ups. All patients were both radiographically and clinically evaluated before the operation and at the final follow-up. The patients’ satisfaction level of the overall outcome was evaluated using the Karlsson-Peterson score, including pain, swelling, instability (subjective), stiffness, stair climbing, running, work activities, and support [17]. Patients’ pain scores were assessed using a visual analogue scale (VAS) [18]. Besides, the Sefton grading system was used to assess ankle joint stability [19].

### Statistical analysis

Patient’s descriptive statistics in categorical variables were reported as number (n) and percentage (%); continuous variables were reported as mean ± standard deviation (SD). Comparisons of the results before the reconstructive operation and at the final follow-up in the radiographic parameters, Karlsson-Peterson, and VAS scores were made by paired t-test. Differences were considered statistically significant when p was less than or equal to 0.05. All statistical analyses were performed using the statistical software package SPSS complex sample module version 22.0 (IBM Corp, Armonk, NY).

## Results

### Patients characteristics

Eighteen patients (15 male/3 female) with chronic ankle instability were included in this study, with the mean age at 27.8 ± 6.0 (range from 20 to 46) years. The majority of patients had occupations that need highly intensive athletic activities such as soldier (*n* = 12), athlete (*n* = 5), and student (*n* = 1). Eight patients had suffered from left side chronic ankle instability and the other 10 patients suffered from the right side. Preoperative stress radiographs on the ankle joint had indicated that the mean talar tilt angle was 25.17 ± 5.28° and the mean anterior talar translation was 10.06 ± 1.80 mm. Thirteencases had grade D to F degrees of damage to the talar cartilage according to the arthroscopic grade system established by Ferkel in 1995, 11 cases had various degrees of anterior ankle impingement, 6 cases had the small avulsion bone fragments on the distal end of the fibula, and 4 cases had os subfibulare.

The average time of follow-up was 33.33 ± 3.69 (range from 24 to 36) months. According to the Sefton Grading System, 94.5% of the patients had excellent/good functional results (10 excellent, 7 good). All 18 patients had been able to resume athletic activities 3 months postoperative, and no recurrence of ankle instability was observed at the final follow-up (Table 1).

**Table 1:** Patients characteristics

Variable	Total, *n* = 18
**Age**	27.83 ± 6.04
**Gender**	
Male	15 (83.3)
Female	3 (16.7)
**Occupation**	
Athlete	5 (27.8)
Soldier	12 (66.7)
Student	1 (5.5)
**Injury side**	
Left	8 (44.4)
Right	10 (55.6)
**Follow-up time (mons)**	33.33 ± 3.69
**Sefton Grading System**	
excellent	10 (55.6)
good	7(38.9)
fair	1 (5.6)

Categorical variables were reported as number (*n*) and percentage (%)

Continuous variables were reported as mean ± SD

**Fig. 7:**
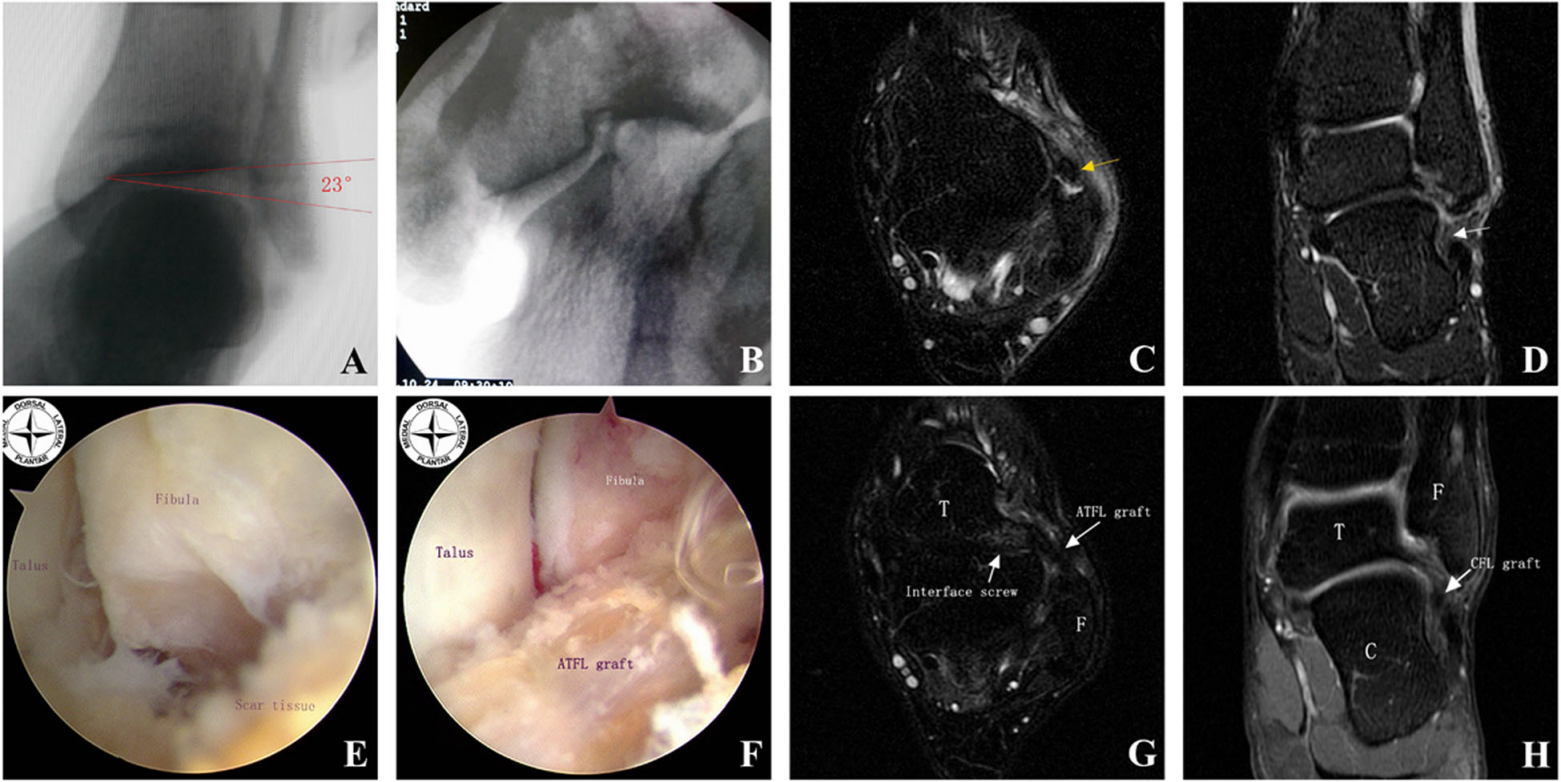
A male patient, age 29, had sprained left ankle joint previously (3 years ago) during practice and subsequently suffered repeated sprains. **a** and **b** The results of stress position fluoroscopy indicated that a talar tilt angle range to 23° with the subtalar joint instability. **c** Sagittal and **d** coronal views of MRI indicated that the structure of the ATFL was damaged (yellow arrow), and CFL was chaotic (white arrow). **e** and **f** Arthroscopic views of the operative site. Severe ligament remnant scarring was found by arthroscopic observation. Suboptimal tendon quality and the semitendinosus tendon transplantation was performed. **g** Sagittal and **h** coronal views of MRI indicating good tendon graft for ligament reconstruction at a follow-up 24 months postoperative. Interface screw, ATFL, and CFL were as indicated (white arrow)

None of the patients suffered from a wound infection or nerve injury. The lateral wall of the fibular bone tunnel was broken in one patient during the surgery while drilling the bone tunnel. A 2.3 mm BR anchor had been reinforced to the tendon graft nearby the entrance of the fibular tunnel. A CT scan at a 3-month follow-up postoperative had shown that the lateral wall of the fibular tunnel had healed well, and the final follow-up had shown no sign of instability or local irritation. There was another patient who suffered from another ankle sprain at postoperative 7 months. Radiography had shown that the break of the posterior fibular cortex was caused by the ENDOBUTTON plate, however, no ankle joint instability was found in the examination. A typical case that had suffered from the recurrence of ankle sprains over 3 y is presented in Fig. 7. With this technique, a good regeneration of the tendon graft was observed after 24-month follow-up.

### Comparison between preoperative and postoperative measurements

At the final follow-up, there was a significant improvement in radiographic parameters, the mean of anterior drawer test decreased from 10.06 ± 1.80 to 3.28 ± 1.02 mm (*p* < 0.001), and talar tilt angle reduced from a preoperative mean of25.17 ± 5.28 to postoperative 4.17 ± 0.92 degrees (*p* < 0.001; Table 2). At the same time, the mean Karlsson-Peterson score was increased from preoperative 52.61 ± 5.48 points to postoperative 91.38 ± 6.60 (*p* < 0.001). This indicated that patients were significantly satisfied with the overall surgery outcomes. The VAS score was improved significantly after the surgery (preoperative: 3.61 ± 1.46 versus postoperative: 0.67 ± 0.59, *p* < 0.001).

**Table 2:** Comparison of Radiographic Parameters, AOFAS and VAS Score

Variable, mean ± SD	Preoperative	Postoperative	*p*-value
**Radiographic measurements**			
Anterior drawer (mm)	10.06 ± 1.80	3.28 ± 1.02	< 0.001
Talar tilt test (degree)	25.17 ± 5.28	4.17 ± 0.92	< 0.001
**Karlsson-Peterson score**	52.61 ± 5.48	91.38 ± 6.60	< 0.001
**VAS score**	3.61 ± 1.46	0.67 ± 0.59	< 0.001

Results were reported as mean ± SD

visual analogue scale (VAS)

## Discussion

A significant association between previous joint injury and resultant long-term chronic ankle instability with the development of posttraumatic osteoarthritis (PTOA) has been reported in the literature [6, 20]. While ligamentous repair or reconstruction has been shown to significantly improve patient satisfaction, symptoms, functions, activity level, and stability, it has not been shown to limit the progression of PTOA [20]^.^ For instance, while the Broström-Gould procedure has been the preferred treatment of chronic ankle instability and offered the advantages of clear-cut effectiveness and few complications, a long-term implication of PTOA has been reported [3, 12, 21]. Vuurberg et al. found that anatomic reconstruction and anatomic repair would provide a better functional outcome than tenodesis reconstruction for patients with chronic ankle instability after surgery [22]. For some patients with long-duration ligament injury, high body mass index (BMI), multiple ligament relaxation, and high exercise requirements, the anatomic reconstruction using a tendon graft was feasible [12]. Our results had shown that anatomic reconstruction of the lateral ankle ligament complex using the arthroscopy combined fluoroscopic technique, leads to improved clinical results and radiographic stability with significant patient satisfaction. In this study, 94.5% of the patients had excellent/good functional results. All patients had been able to resume athletic activities 3 months postoperative, and no recurrence of ankle joint instability was reported at the final follow-up. As it was mentioned above, when performing minimally invasive reconstruction surgery, locating the anatomical points on the ligament proved to be difficult. Most studies that described minimally invasive reconstruction techniques didn’t clarify how anatomical ligament could be reconstructed [14, 15].

Arthroscopic reconstruction provides a direct view of the ligament remnant and enables a successful anatomic reconstruction with a lower amount of operative aggression and minimal disturbance [14]. The arthroscopy not only improves the diagnosis and the management of associated intra-articular lesions, but also control the anatomic positioning of the repair/graft [23]. Guillo et al. in 2014 [24] had first reported an arthroscopic procedure of lateral collateral ankle ligament anatomical reconstruction and Takao et al. had provided a more detailed description of this procedure [25]. In these studies, the tension of the tendon graft was adjusted with the pulley effect technique which has higher requirements. According to the literature, there are many choices to drill the talar tunnel in the ATFL reconstruction; but the creation of a blind tunnel with a diameter of 5 mm and a depth of 20 mm toward the rear is the safest method which had been reported [26].

Matsui et al. reported that the footprint of the ATFL was easy to be pinpointed under arthroscopic examination, but the calcaneal footprint of the CFL located approximately 17 mm perpendicularly below the center point of the rear joint surface of the calcaneus was difficult to be located accurately [15]. The ligament remnant is the most accurate sign of the anatomical location of the bone tunnel entrance. In the present study, the arthroscopic locating of the fibular and talar footprints (ligament insertions) of the ATFL combined with the use of fluoroscopy was employed to locate the calcaneal insertion of the CFL (the ATFL and CFL are mutually adjacent at the fibular insertion). A narrow bare area was found between the anterior edge of the lateraltalar articular surface and the ligament remnant, which can be used as ananatomic mark of the entrance of the talus tunnel. At the same time, the position of the talar and calcaneal guide wire (the location of the entrance of the talar and calcaneal bone tunnel) was confirmed using fluoroscopy. This study showed that the combination of the arthroscopic and fluoroscopic method was used to obtain an accurate anatomical location of the entrance of the bone tunnels, which was not previously reported.

In the past, patients with chronic ankle instability who were not suitable for ligament repair were treated by using a gracilis tendon autograft via arthroscopic ATFL reconstruction and achieved good preliminary effectiveness [27]. However, this method was not appropriate for the patients with complex ankle instability along with subtalar joint instability (damage to the CFL) and talar tilt angles obtained from varus stress position radiographs of >15° [1, 16] Therefore, the modified arthroscopic procedure of anatomic reconstruction in this study was suitable for patients who had chronic ankle instability with relatively severe ligament remnant scarring and local tissue that is not fit for suture anchor repair and those who had chronic ankle instability with old avulsion fractures at the end of the fibula or ossubfibulare where the tension-free repair can’t be performed after debridement.

There were limitations in this study. First, the sample size was small. Second, this study was a case series study without a control group. Third, the follow-up time was relatively short, a long-term follow-up should be completed to further evaluate the clinical outcomes. Fourth, the anatomical variation was unable to be considered after we had recited the anatomy data from the literature which used fluoroscopy for positioning at the entrance to the calcaneal tunnel.

## Conclusion

The anatomic reconstruction of the ankle lateral ligament complex to treat chronic ankle instability using the arthroscopy combined with the fluoroscopic technique could improve clinical functions, patient satisfaction, and reduced pain after the operation. A well-designed clinical trial is needed to confirm the effectiveness of using this modified arthroscopic procedure of anatomic reconstruction to treat chronic ankle instability in the future.

## Data Availability

The datasets used during the current study are available from the corresponding author on reasonable request.

## Abbreviations

ATFL: Anterior talofibular ligament
CFL: Calcaneofibular ligament
FOT: Fibular obscure tubercle
AM: Anteromedial
AL: Anterolateral portal
TLC: Tuberculum ligament calcaneofibular
IL: inferolateral

## References

[1] Noailles T, Lopes R, Padiolleau G, Gouin F, Brilhault J (2018). Non-anatomical or direct anatomical repair of chronic lateral instability of the ankle: a systematic review of the literature after at least 10 years of follow-up. Foot Ankle Surg..

[2] Simon JE, Docherty CL (2018). Health-related quality of life is decreased in middle-aged adults with chronic ankle instability. J Sci Med Sport.

[3] Brown CN, Rosen AB, Ko J (2015). Ankle ligament laxity and stiffness in chronic ankle instability. Foot Ankle Int.

[4] Doherty C, Delahunt E, Caulfield B, Hertel J, Ryan J, Bleakley C (2014). The incidence and prevalence of ankle sprain injury: a systematic review and meta-analysis of prospective epidemiological studies. Sports Med.

[5] Sugimoto K, Isomoto S, Samoto N, Okahashi K, Araki M (2017). Recent developments in the treatment of ankle and Subtalar instability. Open Orthop J.

[6] Al-Mohrej OA, Al-Kenani NS (2016). Chronic ankle instability: current perspectives. Avicenna J Med.

[7] Cao Y, Hong Y, Xu Y, Zhu Y, Xu X (2018). Surgical management of chronic lateral ankle instability: a meta-analysis. J Orthop Surg Res.

[8] Purevsuren T, Batbaatar M, Khuyagbaatar B, Kim K, Kim YH (2018). Comparative evaluation between anatomic and non-anatomic lateral ligament reconstruction techniques in the ankle joint: a computational study. J Biomech Eng.

[9] Hua Y, Chen S, Li Y, Chen J, Li H (2010). Combination of modified Brostrom procedure with ankle arthroscopy for chronic ankle instability accompanied by intra-articular symptoms. Arthroscopy..

[10] de Vries JS, Krips R, Sierevelt IN, Blankevoort L, van Dijk CN. Interventions for treating chronic ankle instability. Cochrane Database Syst Rev. 2011:CD004124. 10.1002/14651858.CD004124.pub3 PMC13254623 21833947

[11] Buerer Y, Winkler M, Burn A, Chopra S, Crevoisier X (2013). Evaluation of a modified Brostrom-Gould procedure for treatment of chronic lateral ankle instability: a retrospective study with critical analysis of outcome scoring. Foot Ankle Surg.

[12] Michels F, Pereira H, Calder J, Matricali G, Glazebrook M, Guillo S (2018). Searching for consensus in the approach to patients with chronic lateral ankle instability: ask the expert. Knee Surg Sports Traumatol Arthrosc.

[13] Xu X, Hu M, Liu J, Zhu Y, Wang B (2014). Minimally invasive reconstruction of the lateral ankle ligaments using semitendinosus autograft or tendon allograft. Foot Ankle Int..

[14] Matsui K, Burgesson B, Takao M, Stone J, Guillo S, Glazebrook M (2016). Minimally invasive surgical treatment for chronic ankle instability: a systematic review. Knee Surg Sports Traumatol Arthrosc.

[15] Matsui K, Burgesson B, Takao M, Stone J, Guillo S, Glazebrook M (2017). Bony landmarks available for minimally invasive lateral ankle stabilization surgery: a cadaveric anatomical study. Knee Surg Sports Traumatol Arthrosc.

[16] Azar FM, Beaty JH, Canale ST. Sports injuries of the ankle. Campbell's Operative Orthopaedics. 13th ed. Philadelphia: Elsevier; 2017.

[17] Karlsson J, Peterson L (1991). Evaluation of ankle joint function: the use of a scoring scale. Foot..

[18] Jensen MP, Karoly P, Braver S (1986). The measurement of clinical pain intensity: a comparison of six methods. Pain..

[19] Sefton GK, George J, Fitton JM, McMullen H (1979). Reconstruction of the anterior talofibular ligament for the treatment of the unstable ankle. J Bone Joint Surg Br.

[20] Blalock D, Miller A, Tilley M, Wang J (2015). Joint instability and osteoarthritis. Clin Med Insights Arthritis Musculoskelet Disord.

[21] Nery C, Raduan F, Del Buono A, Asaumi ID, Cohen M, Maffulli N (2011). Arthroscopic-assisted Brostrom-Gould for chronic ankle instability: a long-term follow-up. Am J Sports Med.

[22] Vuurberg G, Pereira H, Blankevoort L, van Dijk CN (2018). Anatomic stabilization techniques provide superior results in terms of functional outcome in patients suffering from chronic ankle instability compared to non- anatomic techniques. Knee Surg Sports Traumatol Arthrosc.

[23] Takao M, Oae K, Uchio Y, Ochi M, Yamamoto H (2005). Anatomical reconstruction of the lateral ligaments of the ankle with a gracilis autograft: a new technique using an interference fit anchoring system. Am J Sports Med.

[24] Guillo S, Cordier G, Sonnery-Cottet B, Bauer T (2014). Anatomical reconstruction of the anterior talofibular and calcaneofibular ligaments with an all-arthroscopic surgical technique. Orthop Traumatol Surg Res.

[25] Takao M, Glazebrook M, Stone J, Guillo S (2015). Ankle arthroscopic reconstruction of lateral ligaments (ankle anti-ROLL). Arthrosc Tech.

[26] Michels F, Guillo S, Vanrietvelde F, Brugman E, Stockmans F, Ankle instability group (2016). How to drill the talar tunnel in ATFL reconstruction?. Knee Surg Sports Traumatol Arthrosc.

[27] Lan S, Zeng W, Yuan G, Xu F, Cai X, Tang M, Wei S (2020). All-inside arthroscopic anterior Talofibular ligament anatomic reconstruction with a Gracilis tendon autograft for chronic ankle instability in high-demand patients. J Foot Ankle Surg.

